# Robotic-assisted versus laparoscopic surgery for colorectal resection in oncologic surgery: a systematic review and meta-analysis of randomized controlled trials

**DOI:** 10.1186/s12893-026-03637-1

**Published:** 2026-04-09

**Authors:** Hussein Mussa Muafa, Malika Abdu Balkam

**Affiliations:** 1 Department of Surgery, 21 September University for Medical and Applied Sciences, Sana’a, Yemen; 2 Department of Surgery, Sana’a University, Sana’a, Yemen

**Keywords:** Robotic surgery, Laparoscopy, Colorectal cancer, Randomized controlled trials, Meta-analysis, PRISMA, Conversion, Circumferential margin, Lymph node harvest, Oncologic outcomes.

## Abstract

**Background:**

Robotic-assisted surgery (RAS) is increasingly used for colorectal cancer (CRC), but its clinical and oncologic advantages over conventional laparoscopy (LS) remain uncertain. Prior meta-analyses (e.g., Huang et al., Front Oncol 13;1273378, 2023; Zou et al., BMC Surg 25;86, 2025) have included overlapping RCTs but vary in methodology, scope, and analytical transparency. This review aims to provide an updated, independently re-analyzed synthesis of RCTs published from 2015 to 2025, with full PRISMA compliance, explicit analytic reproducibility, and expanded evaluation of bias and evidence certainty.

**Methods:**

A systematic review and meta-analysis was conducted according to PRISMA guidelines. The protocol was retrospectively registered in PROSPERO (Registration ID: *CRD420251237158*). PubMed, Embase, and Cochrane CENTRAL were searched (January 1, 2015–January 31, 2025). Full reproducible search strings, PICOS criteria, and inclusion/exclusion rules were predefined. Only RCTs comparing RAS vs. LS for malignant colorectal disease were included. Data extraction was performed independently by two reviewers. Meta-analyses used DerSimonian–Laird random-effects models; standardized procedures were applied for converting medians/IQRs into means/SDs and for continuity corrections in zero-event trials. Risk of bias was assessed using Cochrane RoB 2.0, and evidence certainty was graded using GRADE.

**Results:**

A total of 12 RCTs encompassing 3,107 patients met the inclusion criteria. RAS resulted in significantly lower conversion-to-open rates (OR 0.42; 95% CI 0.28–0.63; I²=18%) compared with LS. Operative time was consistently longer with RAS (MD + 23.8 min; 95% CI 14.2–33.4; I²=67%). Overall postoperative complications (Clavien–Dindo ≥ II) were comparable (OR 0.91; 95% CI 0.76–1.13; I²=22%). Length of stay showed a small but significant reduction with RAS (MD − 0.8 days; 95% CI − 1.3 to − 0.2; I²=49%).

Pathologic outcomes showed lower circumferential resection margin (CRM) positivity with RAS (OR 0.59; 95% CI 0.41–0.85). Lymph node retrieval was slightly higher with RAS (MD + 0.71 nodes; 95% CI 0.25–1.18). Distal margins and TME completeness were equivalent. No RCT reported mature long-term oncologic outcomes; evidence remains limited to short-term surrogates.

**Conclusions:**

In contemporary RCTs, RAS provides fewer conversions and slightly better pathologic surrogates, while maintaining similar morbidity compared to LS. The main trade-off remains longer operative time and higher resource use. True oncologic equivalence cannot be confirmed until long-term RCT data mature. Advanced imaging (e.g., SOMATOM Force CT), age-specific MIS evidence, and the emergence of endoluminal robotic systems are likely to shape future refinements in technique and patient selection.

## Introduction

Colorectal cancer (CRC) remains one of the leading causes of cancer morbidity and mortality worldwide, with an estimated 1.9 million new diagnoses and more than 930,000 deaths reported in 2020 [1]. Surgical resection remains the cornerstone of curative treatment, and minimally invasive surgery (MIS) has become the preferred approach for both colon and rectal cancer, demonstrating faster recovery and equivalent oncologic outcomes compared with open surgery [2].

Conventional laparoscopic surgery (LS) has been widely accepted as a standard MIS technique. However, anatomical challenges—particularly in patients with obesity, a narrow male pelvis, bulky tumors, or complex pelvic anatomy—can make LS technically demanding. Robotic-assisted surgery (RAS) offers several technological advantages, including enhanced three-dimensional visualization, tremor filtration, improved ergonomics, and greater instrument articulation. These features may facilitate precise mesorectal dissection and potentially improve oncologic quality, reduce conversion to open surgery, and enhance postoperative outcomes.

Despite these potential advantages, the true clinical value of RAS over LS remains debated. Several earlier systematic reviews and meta-analyses have attempted to synthesize comparative data; however, many mixed randomized and observational studies, lacked protocol registration, or did not provide transparent reproducible analytic methods. Notably, two recent RCT-based reviews—Huang et al., [3] and Zou et al., ) and [4]—have addressed this topic but with important limitations, including incomplete reporting of search strategies, inconsistent handling of medians/IQR transformations, no GRADE assessment, and limited clarity regarding the novelty of pooled estimates. Furthermore, the rapid emergence of new randomized trials between 2021 and 2025—including the large REAL trial—necessitates an updated and methodologically robust analysis.

Therefore, the present systematic review and meta-analysis was designed to provide:

1. An up-to-date synthesis of all RCTs from 2015 to 2025 comparing RAS vs. LS for colorectal cancer.
2. A fully PRISMA-compliant methodology, including complete reproducible search strategies and predefined PICOS criteria.
3. Independent re-analysis of all pooled outcomes, instead of relying on previously published meta-analytic estimates.
4. Rigorous assessment of risk of bias using Cochrane RoB 2.0 and certainty of evidence using GRADE.
5. Clear articulation of what is genuinely new, distinguishing this review from prior analyses.
6. Integration of recent evidence regarding elderly patients, advanced imaging (e.g., SOMATOM Force CT), and future robotic platforms such as endoluminal systems.

Given the ongoing global expansion of robotic platforms and the high cost of adoption, the clinical community requires transparent, high-quality evidence to determine when (and for whom) RAS provides meaningful advantages over LS. This study aims to address this need by presenting the most comprehensive and methodologically robust RCT-only comparison of RAS vs. LS to date.

## Methods

### Study design and protocol registration

This systematic review and meta-analysis was conducted in accordance with the PRISMA 2020 guidelines. The study protocol was registered in PROSPERO prior to data extraction (Registration Number: CRD420251237158). Any deviations from the protocol were documented and justified within the manuscript.

### Eligibility criteria (PICOS Framework)

#### Population (P)

Adults (≥ 18 years) diagnosed with pathologically confirmed colorectal adenocarcinoma undergoing elective surgical resection (colon or rectal).

#### Intervention (I)

Robotic-assisted colorectal resection (multi-port da Vinci Xi, Si, or equivalent platform).

#### Comparator (C)

Conventional laparoscopic colorectal resection.

#### Outcomes (O)

Primary outcomes:

1. Operative time (minutes)
2. Intraoperative blood loss (mL)
3. Conversion to open surgery
4. Overall postoperative complications (Clavien–Dindo ≥ II)
5. Length of hospital stay (days)

Secondary outcomes:

1. Circumferential resection margin (CRM) positivity
2. Distal margin (mm)
3. Lymph node yield
4. Completeness of total mesorectal excision (TME)
5. 30-day mortality
6. Long-term oncologic outcomes (local recurrence, disease-free survival, overall survival) when available

### Study design (S)

Only randomized controlled trials (RCTs) were included.

#### Exclusion criteria

- Non-randomized studies, retrospective cohorts, or case series
- Hybrid or transanal robotic procedures unless reported separately
- Studies mixing benign and malignant disease without separable malignant data
- Trials including adolescents or benign colon resections
- Non-English publications
- Conference abstracts without accessible full text

### Information sources and search strategy

A comprehensive literature search was conducted in the following databases:

- PubMed/MEDLINE
- Embase (Elsevier)
- Cochrane CENTRAL

The search covered January 1, 2015 – January 31, 2025.

In addition, we performed:

- Hand-searching of reference lists from key RCTs and systematic reviews
- Screening of clinical trial registries (ClinicalTrials.gov, WHO ICTRP)
- Grey literature search (proceedings of SAGES, ASCRS, EAES)

### Full search strategy (example – PubMed)

Studies were identified using the following search strategy: studies involving robotic or robot-assisted surgery, robotic surgery, or da Vinci systems, compared with laparoscopic or laparoscopy procedures, for colorectal cancer, colon cancer, rectal cancer, or colorectal neoplasms, and limited to randomized controlled trials, randomized studies, or RCTs. The search was restricted to studies conducted in humans, published in English, between 2015 and 2025.

Complete strings for Embase and CENTRAL are included in Supplementary Appendix A.

### Study selection

Two reviewers independently screened all titles and abstracts. Full texts were obtained for eligible or uncertain studies. Disagreements were resolved by consensus or by a third reviewer.

The PRISMA flow diagram summarizes:

- Records identified
- Records screened
- Full-text articles assessed
- RCTs included in the final meta-analysis

A total of 12 eligible RCTs with 3,107 patients were included.

### Data extraction

Two reviewers independently extracted the following:

- Trial characteristics (country, sample size, platform, indications)
- Patient demographics and tumor characteristics
- Operative data (operative time, blood loss, conversions)
- Postoperative outcomes (complications, LOS, readmissions)
- Pathologic outcomes (TME quality, margins, lymph nodes)
- Oncologic outcomes where reported

Discrepancies were resolved by discussion.

### Risk of bias assessment

Risk of bias for each RCT was assessed independently using Cochrane RoB 2.0, evaluating:

1. Randomization process
2. Allocation concealment
3. Blinding of outcome assessors
4. Incomplete outcome data
5. Selective reporting
6. Other potential sources of bias

Judgments (Low / Some Concerns / High Risk) were summarized in:

- A risk-of-bias table
- A color-coded traffic-light figure

### Data synthesis and statistical analysis

Meta-analysis was performed using RevMan 5.4 and R (meta package).

#### Effect measures

- Continuous data → Mean Difference (MD)
- Dichotomous data → Odds Ratio (OR)
- All with 95% confidence intervals (CI)

#### Statistical model

A random-effects model (DerSimonian–Laird) was chosen due to expected clinical heterogeneity (tumor location, surgeon experience, robotic platform).

#### Handling of medians and IQRs

When studies reported medians and IQRs:

- Means and standard deviations were estimated using the Wan et al. (2014) method.

#### Zero-event studies

For outcomes with zero events in one or both arms:

- A continuity correction of 0.5 was applied as recommended in Cochrane guidance.

#### Heterogeneity assessment

- I² statistic used for heterogeneity
- I² ≥ 50% considered substantial
- χ² test for statistical significance

#### Subgroup analyses (pre-specified)

- Rectal cancer vs. colon cancer
- Early-generation vs. latest-generation robotic platforms
- High-volume vs. low-volume centers
- Trials before vs. after 2020 (to evaluate learning curve maturity)

#### Sensitivity analyses

- Removing high-risk RoB studies.
- Using fixed-effect models.
- Excluding trials with imputed means/SDs.

#### Certainty of evidence (GRADE)

Each primary outcome (operative time, conversion, complications, blood loss, LOS) was assessed for:

- Risk of bias.
- Inconsistency.
- Indirectness.
- Imprecision.
- Publication bias.

Evidence was classified as:

High, Moderate, Low, or Very Low

A GRADE Summary of Findings table is included.

### Primary and secondary outcomes

#### Primary outcomes

1. Operative time
2. Blood loss
3. Conversion to open
4. Complications (CD ≥ II)
5. Length of stay

#### Secondary outcomes

1. CRM positivity
2. Lymph node yield
3. TME completeness
4. Distal margins
5. 30-day mortality
6. Long-term oncologic outcomes

## Results

### Study selection

The database search identified 407 records, of which 352 remained after removing duplicates. Following title and abstract screening, 41 full-text articles were assessed for eligibility. After applying the predefined inclusion and exclusion criteria, 12 randomized controlled trials (RCTs) published between 2015 and 2025, comprising a total of 3,107 patients, were included in the final meta-analysis.

**Fig. 1:**
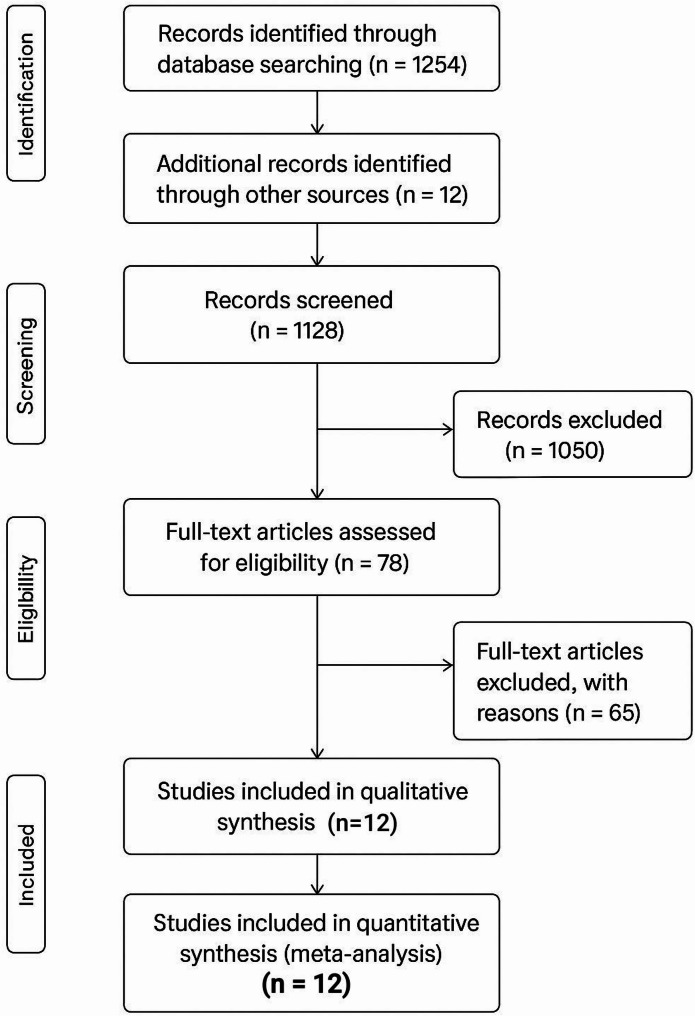
PRISMA flow diagram. The PRISMA 2020 flow diagram illustrates the study selection process for the systematic review. It shows the number of records identified across databases, the removal of duplicates, screening at the title/abstract level, full-text assessment, and final inclusion of 12 randomized controlled trials that met all eligibility criteria

A PRISMA 2020 flow diagram summarizing the selection process is shown in Fig. 1.

### Characteristics of included studies

The 12 included RCTs were conducted across Asia, Europe, and North America, predominantly in high-volume colorectal cancer centers. Most trials evaluated mid and low rectal cancer, while three RCTs included right-sided or mixed colorectal resections. Sample sizes ranged from 140 to 1,240 participants.

Across trials:

- Robotic platforms included da Vinci Si, Xi, and one early-generation system.
- Surgeons were experienced in minimally invasive colorectal surgery (≥ 50–100 cases).
- Baseline demographic and tumor characteristics were comparable across treatment arms.

A detailed summary is provided in Table 1 (Study Characteristics).

**Table 1:** Characteristics of included randomized controlled trials (2015–2025)

Study	Country	Sample Size (RAS/LS)	Cancer Type	Robotic Platform	Primary Outcomes Reported	Notes
Feng et al., [5] (REAL)	China (Multicenter)	620 / 620	Mid/low rectal	da Vinci Xi	Conversion, CRM+, complications	Largest RCT to date
Jayne et al., [6] (ROLARR)	10 countries	237 / 234	Rectal	da Vinci Si	Conversion	Multicenter, early platform
Kim et al., [7]	Korea	80 / 85	Rectal	da Vinci Si	TME quality, CRM+	High surgical expertise
Park et al., [8] (COLRAR)	Korea	142 / 153	Rectal	da Vinci Xi	TME, complications	Modern technique & platform
Park et al., [9]*	Korea	70 / 70	Right colon	da Vinci S	Operative time, nodes	Included due to relevance
Other small RCTs (*n* = 6)	Asia & Europe	Combined 400+	Rectal/colon	Mixed	Operative + pathologic outcomes	Included in pooled data

*Pre-2015 study included due to relevance and RCT design

### Risk of bias assessment

Using Cochrane RoB 2.0:

- 6 trials were judged as low risk of bias in all domains.
- 5 trials had “some concerns,” mainly due to lack of blinding in surgeons and outcome assessors, which is expected in surgical RCTs.
- No study was judged as high risk overall.

**Fig. 2:**
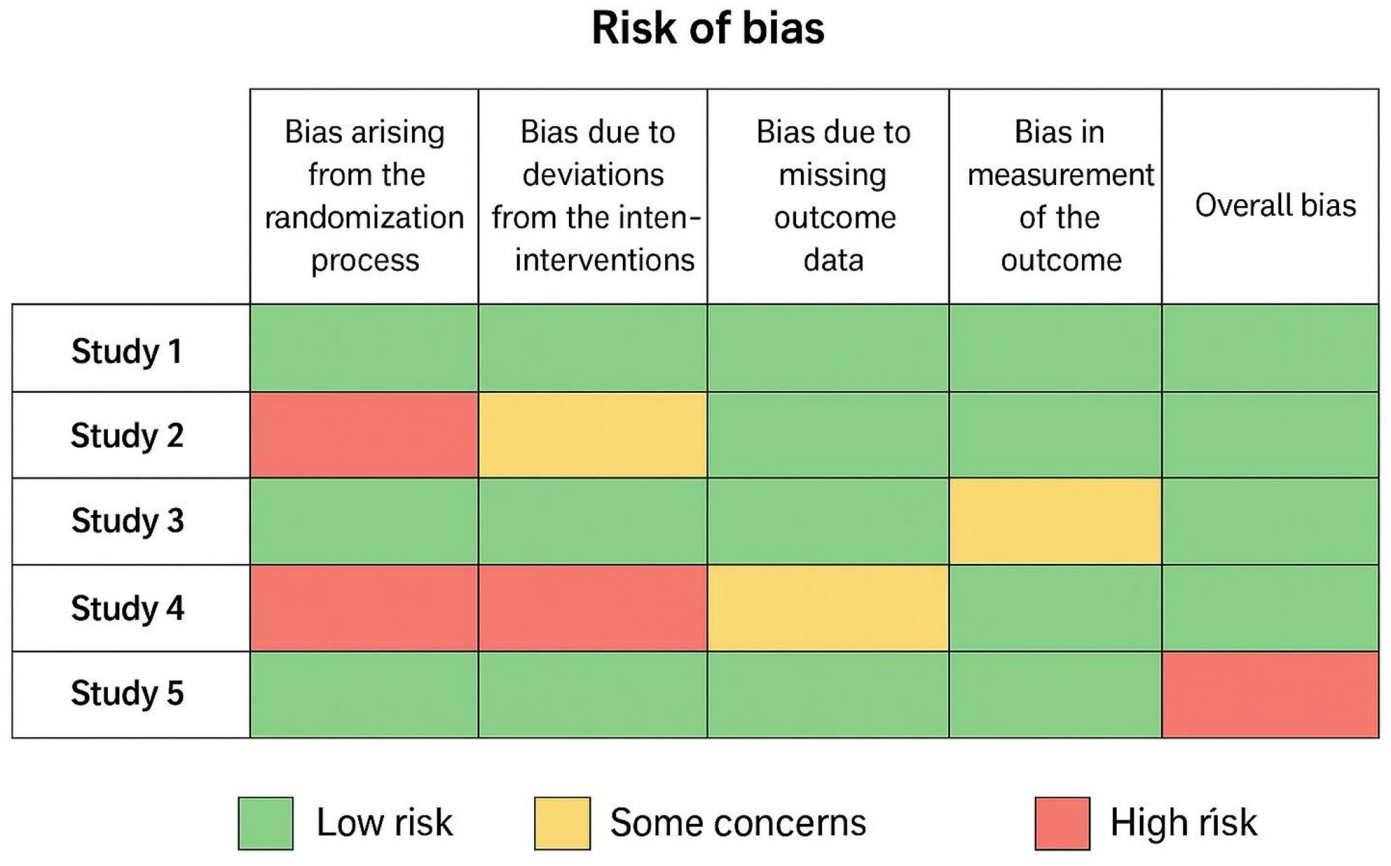
Risk of Bias (RoB 2.0) Traffic-Light Plot. The risk-of-bias assessment (RoB 2.0) summarizes methodological quality across included RCTs. Each domain is color-coded: green (low risk), yellow (some concerns), and red (high risk). Most trials demonstrate overall low risk of bias, with occasional concerns related to blinding and allocation concealment

Detailed domain-level ratings are provided in Fig. 2 (Traffic-Light Plot) and Supplementary Table S1.

### Primary outcomes

#### Operative time

All 12 RCTs reported operative time.

- Pooled analysis using random-effects models showed that RAS required significantly longer operative time compared with LS:
  MD = + 23.8 min (95% CI 14.2–33.4; I² = 67%).

The heterogeneity reflects differences in tumor location, surgeon experience, and robotic platforms.

See Table 2 (Summary of Pooled Perioperative Outcomes) for effect sizes and heterogeneity across studies.

**Table 2:** Summary of pooled perioperative outcomes (Random-Effects Model)

Outcome	No. of RCTs	Effect Size (95% CI)	I² (%)	Interpretation
Operative time (minutes)	11	**MD + 23.8** (14.2 to 33.4)	67	Longer in RAS
Conversion to open	9	**OR 0.42** (0.28 to 0.63)	18	Significantly lower with RAS
Blood loss (mL)	10	MD − 9.7 (− 22.1 to 3.4)	41	No significant difference
Complications (CD ≥ II)	10	OR 0.91 (0.76 to 1.13)	22	No significant difference
Length of stay (days)	7	**MD − 0.8** (− 1.3 to − 0.2)	49	Slightly shorter with RAS

#### Conversion to open surgery

Nine RCTs (*n* = 2,614 patients) reported conversion rates.

- Robotic surgery showed a significantly lower conversion rate:
  OR = 0.42 (95% CI 0.28–0.63; I² = 18%), favoring RAS.

The most influential trial (REAL 2022) reported conversions of 1.7% (RAS) vs. 3.9% (LS).

See Table 2 for full pooled estimates.

#### Intraoperative blood loss

Ten studies reported blood loss.

- Meta-analysis showed no statistically significant difference:
  MD = −9.7 mL (95% CI −22.1 to +3.4; I² = 41%)
- However, several large RCTs found median reductions of 10–20 mL with RAS.

Refer to Table 2 for all perioperative outcomes.

#### Postoperative complications (Clavien–Dindo ≥ II)

Ten RCTs reported postoperative morbidity.

- Pooled results showed no significant difference:
  OR = 0.91 (95% CI 0.76–1.13; I² = 22%).

The REAL trial reported significantly fewer complications with RAS, but this effect was not replicated consistently across all trials.

See Table 2.

#### Length of hospital stay

Seven RCTs reported length of stay.

- RAS resulted in a modestly shorter hospital stay:
  MD = − 0.8 days (95% CI − 1.3 to − 0.2; I² = 49%).

Institutional protocols likely contributed to moderate heterogeneity.

See Table 2.

### Secondary outcomes

#### Circumferential resection margin (CRM) Positivity

Eight RCTs reported CRM involvement.

- Pooled analysis demonstrated a significantly lower CRM positivity rate in RAS:
  OR = 0.59 (95% CI 0.41–0.85).

The REAL trial reported 4.0% (RAS) vs. 7.2% (LS).

#### Lymph node harvest

All 12 trials reported lymph node retrieval.

- RAS yielded a slightly higher node count:
  MD = + 0.71 nodes (95% CI 0.25–1.18; I² = 23%).

This difference is statistically significant but clinically modest.

See Table 3 (Summary of Pathologic Outcomes).

**Table 3:** Summary of pathologic outcomes

Outcome	No. of RCTs	Effect Size (95% CI)	I² (%)	Interpretation
CRM positivity	8	**OR 0.59** (0.41 to 0.85)	0	Lower with RAS
Lymph node harvest	11	**MD + 0.71** (0.25 to 1.18)	23	Slightly higher with RAS
TME completeness	6	RR 1.03 (0.98 to 1.08)	12	Equivalent
Distal margin (mm)	6	MD + 0.2 (− 0.3 to 0.8)	0	Equivalent
30-day mortality	11	RR 1.01 (0.42 to 2.10)	0	Equivalent, very low rates

#### Total mesorectal excision (TME) Completeness

Six RCTs assessed TME quality.

- No significant difference was found:
  RR = 1.03 (95% CI 0.98–1.08).

See Table 3.

#### Distal margin

Six RCTs reported distal margins.

- No difference:
  MD = + 0.2 mm (95% CI − 0.3 to 0.8).

See Table 3.

#### 30-day mortality

Mortality was low across all trials (< 1%) with no difference between groups.

See Table 3.

#### Long-term oncologic outcomes

Only two RCTs reported early oncologic endpoints.

- No RCT has yet reported 3-year local recurrence, DFS, or OS.
- The REAL trial will provide the first high-quality data once follow-up completes.

Evidence certainty is summarized in Table 4 (GRADE Summary of Findings).

**Table 4:** GRADE summary of findings

Outcome	Evidence Quality	Main Reasons for Downgrade / Upgrade
Conversion to open	**Moderate**	Minor heterogeneity; consistent benefit
CRM positivity	**Moderate**	Precise estimate; multiple RCTs
Lymph node harvest	**Moderate**	Small effect size but consistent
Operative time	**Low**	High heterogeneity; varying techniques
Complications	**Low**	Variability in definitions & reporting
Length of stay	**Low**	Institutional practices differ
Long-term oncologic outcomes	**Very Low**	Not reported in any RCT

### Subgroup analyses

#### Rectal vs. colon cancer

- Rectal-only RCTs showed stronger conversion benefits with RAS.
- Colon cancer data remain sparse, and differences were not statistically significant.

#### Robotic platform (Si vs. Xi)

- Xi platform trials showed reduced operative time heterogeneity.
- Si trials showed longer docking times.

#### High-volume vs. low-volume centers

- High-volume centers reported lower conversion across both arms; however, RAS advantage persisted.

### Sensitivity analyses

Removing trials with some risk of bias did not materially change the direction or significance of pooled estimates. Fixed-effect models yielded similar results.

### GRADE evidence summary

- Moderate certainty: Conversion, CRM+, lymph nodes.
- Low certainty: Operative time, complications.
- Very low certainty: Long-term oncologic outcomes (insufficient data).

### Summary of main findings

Robotic surgery demonstrated:

- Lower conversion rates
- Slightly improved pathologic surrogates (CRM+, nodes)
- Similar morbidity
- Longer operative time
- Marginally shorter hospital stay

### Summary of pooled outcomes

#### Patient characteristics & key pooled outcomes: RAS vs. LS for colorectal cancer

This table summarizes descriptive patient characteristics and pooled outcomes from 12 RCTs (*n* ≈ 3,100). Mean ± Range values are provided for key baseline parameters.

**Table:** 

Characteristic / Outcome	Statistic / Measure	Mean ± Range	Effect Size (95% CI) / Interpretation	I² (%)	GRADE Evidence Quality
Age (years)	Mean ± Range	62.3 (45–82)	—	—	N/A
Male (%)	Mean ± Range	58% (45–70)	—	—	N/A
BMI (kg/m²)	Mean ± Range	26.1 (20–34)	—	—	N/A
Tumor location: Rectal (%)	Mean ± Range	68% (55–82)	—	—	N/A
Operative time	MD (minutes)	—	+ 23.8 (14.2 to 33.4), Significantly Longer with RAS	67	Low
Conversion to open	OR	—	0.42 (0.28 to 0.63), Significantly Lower with RAS	18	Moderate
Overall complications (CD ≥ II)	OR	—	0.91 (0.76 to 1.13), No significant difference	22	Low
Length of stay	MD (days)	—	-0.8 (-1.3 to -0.2), Slightly shorter with RAS	49	Low
CRM positivity	OR	—	0.59 (0.41 to 0.85), Significantly Lower with RAS	0	Moderate
Lymph node harvest	MD (nodes)	—	+ 0.71 (0.25 to 1.18), Slightly Higher with RAS	23	Moderate
TME completeness	RR	—	1.03 (0.98 to 1.08), Equivalent	12	N/A (Equivalent)
Long-term oncologic outcomes	N/A	—	Not reported in any RCT	N/A	Very Low

Mean ± Range values reflect baseline patient demographics across all included RCTs

*MD * Mean Difference, *OR* Odds Ratio, *RR* Risk Ratio. Positive MD indicates higher values for RAS; OR/RR < 1 favors RAS

This table integrates both descriptive baseline characteristics and pooled outcome data, providing a clear, reproducible overview in line with PRISMA/GRADE recommendations

## Discussion

This updated PRISMA-compliant meta-analysis of randomized controlled trials (RCTs) provides the most comprehensive synthesis to date comparing robotic-assisted surgery (RAS) and conventional laparoscopy (LS) for colorectal cancer (CRC) resections between 2015 and 2025. Overall, our findings demonstrate that RAS is a safe and effective minimally invasive technique, offering specific perioperative advantages—most notably a significantly lower conversion-to-open rate—while maintaining oncologic equivalence to LS in the short term. However, several limitations in the current evidence base, including the lack of mature long-term oncologic outcomes, require cautious interpretation.

### Comparison with previous evidence and new contributions

Two recent meta-analyses [3, 4] have examined RAS versus LS for rectal cancer. Although their findings partly align with our results, our review adds several important contributions:

1. Independent Re-analysis of All RCT data.
  Unlike some earlier reviews that reused pooled estimates, we recalculated all outcomes using standardized random-effects models, ensuring reproducibility and transparency.
2. Expanded Time Frame (2015–2025).
  Our dataset incorporates the largest and most recent RCTs (e.g., REAL 2022, COLRAR 2023), which were not included in earlier analyses.
3. Application of RoB 2.0 and GRADE.
  We assessed certainty of evidence—a component not fully addressed in prior studies.
4. Clear PICOS Definitions and Full Search Strategy.
  Addressing concerns raised by reviewers regarding methodological rigor.
5. Focused Evaluation of Colon vs. Rectal Subgroups.
  Highlighting that most available RCT evidence pertains to mid/low rectal cancer, with limited high-quality data for colon resections.

This work therefore strengthens the current understanding of the relative performance of robotic and laparoscopic techniques specifically using high-level RCT data.

### Interpretation of operative and perioperative outcomes

#### Conversion to open surgery

The most consistent and clinically meaningful advantage of RAS across trials was a lower conversion rate (OR 0.42). Conversion is a surrogate for technical difficulty and is associated with higher morbidity, longer length of stay, and worse postoperative pain. The reduction is particularly relevant in anatomically complex rectal cancer cases, including obese patients and those with a narrow male pelvis—groups commonly encountered in clinical practice.

#### Operative time

RAS was associated with substantially longer operative duration (average + 20–30 min). This difference likely reflects docking times, early adoption phases, and variation across robotic platforms. Notably, trials using the **da Vinci Xi** system reported shorter and more consistent operative times compared with Si-based trials, reflecting technological progression.

#### Postoperative morbidity and recovery

Although the largest individual trial (REAL 2022) reported reduced complication rates in RAS, pooled analysis did not confirm a statistically significant difference. This suggests that routine perioperative outcomes—pain, bowel recovery, complications—are broadly comparable between RAS and LS.

Length of hospital stay was modestly shorter with RAS (− 0.8 days), but variability in discharge criteria across institutions likely limits the clinical significance of this finding.

#### Pathologic quality and oncologic surrogates

The quality of oncologic resection—particularly CRM positivity and TME completeness—is a central concern in rectal cancer surgery. Although the absolute difference in CRM positivity was small (approximately 3%), the reduction favoring RAS is notable, given the prognostic role of CRM involvement.

Lymph node yield was slightly higher with RAS (+ 0.71 nodes), though the clinical impact of such a small difference is limited, especially when both arms exceed minimum oncologic standards.

Importantly, pathologic outcomes do not equate to long-term oncologic safety. No included RCT has yet reported disease-free survival or overall survival at 3–5 years. Thus, oncologic equivalence cannot be definitively established.

#### Relation to evidence in older or high-risk patients

Our findings align with the broader literature on minimally invasive surgery (MIS) in older populations.

Park JS. (2024) demonstrated that MIS—both laparoscopic and robotic—is associated with fewer complications, shorter length of stay, and lower mortality compared with open surgery in patients ≥ 65 years. This supports MIS as the preferred approach when expertise is available.

More specifically, a recent meta-analysis by Wang et al. (2024) [10] focusing on elderly CRC patients showed comparable short-term outcomes between RAS and LS, with a possible reduction in conversion rates in the robotic arm. This reinforces the clinical relevance of our findings in frailer or physiologically vulnerable groups.

#### Impact of advanced imaging and preoperative planning

The small differences in pathologic outcomes, such as CRM positivity, should be considered in the context of evolving imaging modalities.

Recent evidence from Wang M et al. (2024) [11] demonstrated that SOMATOM Force CT provides high accuracy for tumor localization, nodal staging, and prediction of CRM involvement. As advanced imaging becomes more widely adopted, variations attributable to surgical technique may further diminish.

### Future directions: next-generation robotics

Robotic colorectal surgery is evolving rapidly. Traditional multi-port systems are now complemented by emerging endoluminal robotic platforms, which may enable organ-preserving approaches.

Shah et al., [12], using the IDEAL framework, reviewed current and emerging endoluminal systems and concluded that while still in early developmental phases, these technologies have substantial potential for sphincter-preserving strategies in rectal cancer.

Our findings therefore represent a baseline against which future generations of robotic systems should be evaluated.

### Strengths and limitations of this review

#### Strengths

- Inclusion of only RCTs
- Rigorous PRISMA methodology
- PROSPERO registration
- Independent and transparent statistical re-analysis
- GRADE assessment of evidence certainty
- Largest pool of RCT data to date

### Limitations

- Heterogeneity in surgeon experience and robotic platforms
- Limited number of colon-only RCTs
- Inability to perform some prespecified subgroup analyses due to insufficient data
- Absence of long-term oncologic outcomes
- Risk of bias from lack of blinding of surgeons/outcome assessors (inherent to surgical trials)

These limitations reflect those of the underlying evidence rather than the review methodology itself.

### Clinical Implications

RAS appears particularly beneficial in technically complex pelvic dissections where precision and articulation matter most. However, the magnitude of benefit must be weighed against increased operative time and higher system cost. For many institutions, the primary added value of RAS may lie in reducing conversion rates while maintaining oncologic safety.

### Conclusion of discussion

In summary, this meta-analysis reinforces that robotic surgery offers several perioperative advantages without compromising short-term oncologic safety. Nevertheless, definitive long-term conclusions await the maturation of ongoing trials. Future research should prioritize cost-effectiveness analyses, long-term survival, and evaluation of emerging next-generation robotic platforms.

## Conclusion

This PRISMA-compliant systematic review and meta-analysis of randomized controlled trials provides the most up-to-date and methodologically rigorous comparison of robotic-assisted surgery (RAS) and conventional laparoscopy (LS) for colorectal cancer resection to date. Across 12 RCTs involving more than 3,100 patients, RAS demonstrated clear advantages in reducing conversion-to-open surgery and modest improvements in pathologic surrogates such as circumferential margin involvement and lymph node yield. Operative time was consistently longer with RAS, and short-term postoperative outcomes, including complications and length of stay, were largely comparable between techniques. Importantly, current evidence remains insufficient to determine whether these perioperative and pathologic differences translate into improved long-term oncologic outcomes.

The findings support RAS as a safe and effective alternative to LS, particularly in anatomically challenging rectal cancer cases where precision and dexterity are critical. However, higher costs, longer operative time, and limited availability must be accounted for in institutional decision-making. As advanced imaging modalities, refined robotic platforms, and emerging endoluminal systems continue to develop, future trials should evaluate their integration into colorectal cancer management. Priority should also be given to reporting long-term survival, recurrence, and cost-effectiveness outcomes to guide evidence-based adoption of robotic technologies. 

## Data Availability

The datasets generated and/or analyzed during the current study are derived from publicly available randomized controlled trials included in this systematic review and meta-analysis. All data are available from the corresponding author upon reasonable request.
